# Temporal Dynamics of Social Anxiety and Depressive Symptoms: The Moderating Role of Cognitive Flexibility

**DOI:** 10.1155/da/3055803

**Published:** 2025-09-15

**Authors:** Reut Zabag, Daniella Mouadeb, Shilat Haim-Nachum, Einat Levy-Gigi, Eva Gilboa-Schechtman

**Affiliations:** ^1^Department of Psychology, Bar-Ilan University, Ramat-Gan, Israel; ^2^Department of Psychology, Yale University, New Haven, Connecticut, USA; ^3^Department of Psychiatry, Anxiety Disorders Program, University of São Paulo, São Paulo, Brazil; ^4^Department of Psychiatry, Columbia University Irving Medical Center, New York, New York, USA; ^5^Faculty of Education, Bar-Ilan University, Ramat-Gan, Israel; ^6^Gonda Multidisciplinary Brain Center, Bar-Ilan University, Ramat-Gan, Israel

**Keywords:** cognitive flexibility, depression, longitudinal design, social anxiety

## Abstract

Social anxiety (SA) and depressive symptoms frequently coexist, with the onset of SA typically preceding the onset of depression. Individuals experiencing SA-depression comorbidity exhibit increased functional impairments as compared to individuals without this comorbidity. Understanding the mechanisms that underlie the coexistence of SA and depressive symptoms can deepen our theoretical understanding regarding these conditions and contribute to depression prevention efforts. Recent theories suggest that low cognitive flexibility (CF) contributes to the development of depression and may play a role in the relationship between the disorders. However, empirical prospective findings are sparse. We examined whether CF moderates the link between SA and depressive symptoms in two independent preregistered studies: cross-sectional (*n* = 379) and longitudinal (*n* = 108, 2-year). Cross-sectionally, higher CF was linked to a weaker relationship between SA and depressive symptoms. Across time, among individuals with low CF at baseline, there was a positive association between SA at baseline and depressive symptoms 2 years later. No such association was found among individuals with high CF. These findings highlight the role of CF in the development of comorbid depressive symptoms among high SA individuals. It may contribute to the clinical prevention of depression through specific psychotherapeutic techniques targeted to enhance CF among SA patients. A preprint of this MS has previously been published.

## 1. Introduction

Experiencing symptoms of social anxiety (SA), depression, or both is frequent and burdensome [1]. Several prospective studies have established that, in most cases, SA precedes depression and increases the risk for depressive symptoms in all ages [2–4]. In fact, socially anxious individuals are five times more likely to develop depression than nonsocially anxious individuals [5]. Moreover, among individuals suffering from depression, SA is the most common anxiety disorder [6]. Additionally, in the presence of SA, depression has a greater severity of symptoms, increased duration of episodes, and decreased social and occupational functioning [5, 7, 8]. This comorbidity is also associated with poorer quality of life, increased relapse rates, poorer treatment outcomes, and increased suicidality [9–11].

Although the association between SA and depressive symptoms has been extensively studied (e.g., [1, 6]), there is limited research on the potential factors that might moderate this relationship (see [12] for an exception). Understanding the moderators of the relationship between SA and depressive symptoms is important in several aspects. Theoretically, it can shed light on the shared mechanism that underlies both disorders, highlighting transdiagnostic factors. Clinically, it can assist in identifying the individuals at higher risk for developing depressive symptoms among high SA individuals. Moreover, these high-risk individuals may be targeted in tailored interventions to prevent the development of comorbid depressive symptoms.

One potential mechanism that may underlie the SA-depression comorbidity is cognitive flexibility (CF) [13, 14]. CF is the ability to identify external or internal demands and dynamically update representations and behaviors [15]. A flexible person will abandon a well-known set of responses in favor of new responses that better suit the situation, whereas a rigid person adheres to the same pattern. Recent theories suggest that low CF underlies a variety of psychopathologies, including both SA and depression [13]. Indeed, low CF, and specifically reduced tendency to update beliefs, modify behaviors, and adjust interpretations were associated with elevated SA and depressive symptoms [16–21].

In addition to a concurrent association with depressive symptoms, low CF was suggested to be a vulnerability mechanism in the development of depressive symptoms [22–24]. Indeed, deficits in CF were prospectively associated with enhanced depressive symptoms in both humans and animals [25]. In addition, CF, alongside constructs closely aligned with CF, such as rigid attitudes, moderated the relationship between risk factors associated with depression and the manifestation of depressive symptoms (e.g., [26, 27]). For example, CF was found to moderate the association between childhood trauma and depressive symptoms [26]. Additionally, individuals exhibiting higher levels of CF exhibit a weaker association between the number of previous depressive episodes and current depressive symptoms, in contrast to those with lower CF levels [28].

CF might play a significant role in understanding depressive symptoms among individuals with high SA. The role of CF may be through multiple pathways. CF was suggested to underlie social avoidance, and higher levels of CF were associated with less social avoidance [29, 30]. Because more social avoidance is prospectively associated with elevated depressive symptoms [31, 32], CF may buffer the role of social avoidance. Higher CF was also associated with less rumination [33]. This cognitive process is prospectively predicted by SA [34] and is considered a mechanism underlying depression [35]. Despite the theoretical connections between these constructs, there remains a gap in understanding how CF might function as a protective factor in the SA-depression relationship.

### 1.1. The Present Research

We examined the potential moderation effects of CF on the relationship between SA and depressive symptoms. We were interested in understanding the role of CF both cross-sectionally and prospectively. We predicted that CF moderates the association between SA and depressive symptoms both concurrently and prospectively, such that higher CF is linked to a weaker relationship between SA and depressive symptoms. Two independent studies were conducted. Study 1 examined the cross-sectional associations between SA, depressive symptoms, and CF. Study 2 prospectively examined the association between SA and depressive symptoms using a 2-year longitudinal design. The hypothesis, data analysis, and data reduction approaches were preregistered (https://osf.io/z6fh3; https://osf.io/wx27u).

## 2. Study 1

### 2.1. Method

#### 2.1.1. Power Analysis

Sample sizes were calculated using the G^*⁣*^*∗*^^Power software [36]. Based on effect sizes found in previous studies examining the role of CF moderating risk factors for depressive symptoms [26–28], we expected to observe a small-medium-sized effect. A-priori power analysis for moderation was conducted to detect this effect size with a significance (*α*) of 5% and power (1 − *β*) of 85%. This analysis suggested the need to recruit at least 300 participants.

#### 2.1.2. Participants

We recruited 472 participants through Amazon's Mechanical Turk (MTurk). MTurk provides an online crowdsourcing platform with access to large and diverse samples suitable for clinical research, collecting mental health data [37]. Moreover, MTurk participants endorse higher SA and depressive symptoms compared to other nonclinical samples [38]. Inclusion criteria for the study were: being 18 years or older, being a resident of the United States, and having high-quality work on previous MTurk tasks (i.e., an acceptance ratio ≥ 95%). Participants were excluded due to IP addresses located outside the United States or associated with U.S.-based virtual private servers (*n* = 23), duplicate IP addresses (*n* = 12), or nonconscientious performance (answer questions at a rate of less than 1 s per item; filling in all items of the questionnaires, including the reversed items, with zero standard deviation; fail in attention check; *n* = 58): Recent research suggests this exclusion method increases data quality [39]. A total of 379 participants were included in the final analysis. Demographic characteristics are presented in Table 1.

#### 2.1.3. Procedure

All participants provided informed consent before the beginning of the study. Participants filled out the self-report questionnaires randomly through a secure research software service (Qualtrics). At the end of the study, participants were debriefed and compensated for their participation. Participants were recruited from October 2020 to January 2021. The Bar-Ilan University Ethics Committee approved the research.

#### 2.1.4. Measures

##### 2.1.4.1. Liebowitz SA Scale

Self-Report version (LSAS-SR; [40]) Consists of 24 items that assess levels of anxiety and avoidance in social or performance situations using a 0–3 Likert-type scale. Higher scores indicate greater levels of anxiety. Reliability scores are presented in Table 1.

##### 2.1.4.2. The Beck Depression Inventory (BDI-II; [41])

A 20-item measure of the severity of depression symptoms in the preceding 2 weeks. Due to ethical concerns, the suicide item (question number 9) was not presented in the online sample. Higher scores indicate greater depressive symptoms.

##### 2.1.4.3. CF

The self-report CF Scale (CFS) [42] is a 12-item measure that assesses the self-perceived ability to communicate effectively, particularly in new situations. Higher scores reflect higher levels of reported CF.

### 2.2. Results

#### 2.2.1. Data Analysis

First, we conducted zero-order correlations on the associations between age, education, SA, CF, and depressive symptoms. Later, we conducted a moderation analysis (Model 1) using the PROCESS macro for R. Based on statistical guidelines [43, 44], independent measures were centered prior to the analysis. Full data and code are available at: https://osf.io/f93xz/?view_only=ef9d73087a744c189019cda0fe2dbec6.

#### 2.2.2. Zero-Order Correlations

Results are presented in Table 2. As can be seen from the Table 2, SA and depressive symptoms were positively moderately associated, while CF was moderately inversely associated with both SA and depressive symptoms.

#### 2.2.3. Hierarchical Multiple Linear Regression

**Figure 1:**
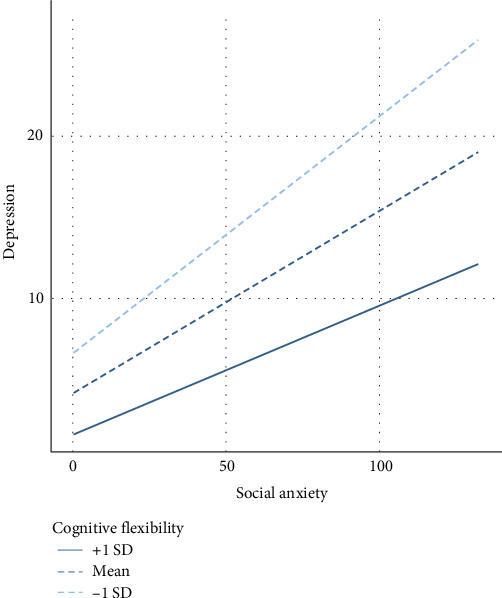
The moderating role of cognitive flexibility in the cross-sectional relationship between social anxiety and depressive symptoms, Study 1.

Table 3 presents a hierarchical multiple linear regression of SA symptoms, CF, and their interaction on depressive symptoms. Results supported our hypothesis, suggesting that CF moderated the relationship between SA symptoms and depressive symptoms (*F*(1375)=4.07, *p*=0.044). Subsequent simple slopes analyses indicated that for individuals reporting low levels of CF (Figure 1, dashed line with short dashes), higher levels of SA were positively associated with higher levels of depressive symptoms (*β*=0.37, *t*=5.91, *p* < 0.001). In contrast, for individuals reporting high levels of CF (Figure 1, solid line), this relationship was weaker (*β*=0.19, *t*=2.55, *p*=0.011). Table S1 presents results controlling for age.

## 3. Study 2

The aim of Study 2 was to shed light on the temporal relationship between CF, SA, and depressive symptoms. Specifically, we examined the longitudinal association between SA and CF in predicting depressive symptoms 2 years later.

### 3.1. Methods

#### 3.1.1. Participants

We contacted 203 participants who had taken part in various previous studies conducted in our lab [20, 45, 46]. None of the participants in Study 1 took part in Study 2. Participants were recruited via MTurk. A total of 111 participants completed the study 2 years later. Participants were excluded based on nonconscientious performance (answered the survey in less than 1 s for a question; *n* = 3). A total of 108 participants were included in the final analysis (demographic characteristics are presented in Table 1). Demographic characteristics of participants who took part only at baseline are presented in Table S2. Individuals who participated at both baseline and follow-up did not differ from those who participated only at baseline in their gender, age, education, or SA but reported lower levels of depressive symptoms and higher levels of CF (see Supporting Information).

#### 3.1.2. Procedure

Study 2 followed a procedure similar to that of Study 1. At Time 1, between July and October 2018, participants provided informed consent before the start of the study. They completed self-report questionnaires in a random order and cognitive tasks using a secure research software platform (Qualtrics). At the end of the study, participants were debriefed and compensated for their participation. At Time 2, between August and October 2020, participants were contacted through MTurk, provided informed consent, completed questionnaires, and were then debriefed and compensated for their participation. The Bar-Ilan University Ethics Committee approved the research.

#### 3.1.3. Measures

Identical to Study 1. Reliability scores are presented in Table 1.

### 3.2. Results

#### 3.2.1. Data Analysis

Zero-order correlations were conducted on the associations between age, education, SA symptoms, CF, and depressive symptoms. Later, we conducted a moderation analysis (Model 1) using the PROCESS macro for *r*. Similar to Study 1 (based on [43, 44]), independent measures were centered previous to the analysis. Full data and code are available at: https://osf.io/f93xz/?view_only=ef9d73087a744c189019cda0fe2dbec6.

#### 3.2.2. Zero-Order Correlations

Results are presented in Table 2. As can be seen from the Table 2, SA was positively associated with depressive symptoms and negatively associated with CF, cross-sectionally and longitudinally. CF and depressive symptoms were negatively associated, both at baseline and at the 2-year follow-up.

#### 3.2.3. Hierarchical Multiple Linear Regression

**Figure 2:**
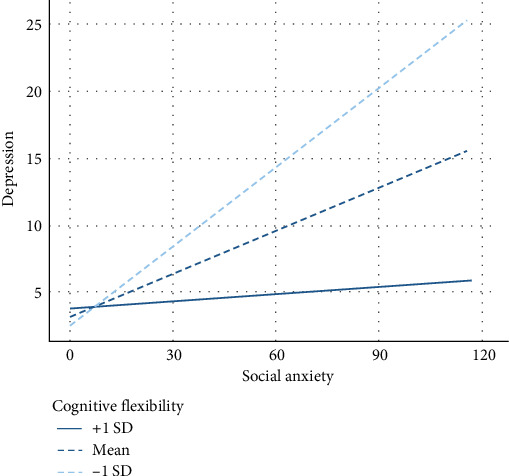
The moderating role of cognitive flexibility in the longitudinal relationship between social anxiety and depressive symptoms, Study 2.

Table 3 presents hierarchical multiple linear regression of SA symptoms and CF at baseline, as well as their interaction in predicting depressive symptoms at follow-up. Results support our hypothesis, suggesting that CF moderated the relationship between SA and depressive symptoms (*F*(1104)=11.81, *p* < 0.001) (Figure 2). Subsequent simple slopes analysis indicated that for individuals reporting low levels of CF (Figure 2, dashed line with short dashes), higher levels of SAwere moderately associated with higher levels of depressive symptoms (*β*=0.55, *t*=5.91, *p* < 0.001). In contrast, for individuals reporting high levels of CF (Figure 2, solid line), no association between SA and depressive symptoms was found (*β*=0.01, *t*=0.15, *p*=0.88). Table S3 presents results controlling for baseline depressive symptoms. Table S4 presents cross-lag panel model predicting depressive symptoms at follow up from depressive symptoms, SA, CF, and the interaction between SA and CF at baseline.

## 4. Discussion

The present research examined the role of CF as a mechanism underlying the comorbidity of SA and depressive symptoms. In two independent, preregistered studies, we found that CF moderates the association between SA and depressive symptoms. Cross-sectionally, the results reveal a partial moderation effect: better CF weakens the relationship between symptoms of SA and depressive symptoms. Longitudinally, the results reveal a full moderation effect: among individuals who report themselves as high in CF, no association between SA and depressive symptoms was found. Thus, our findings suggest that high CF may act as a buffer between SA symptoms and the development of depressive symptoms.

The current research highlights the role of CF as an aspect of resilience [47, 48]. Indeed, high CF appears to buffer against negative experiences, thus mitigating the development of other psychopathologies [49]. For instance, CF moderated the relationship between work-related stress and general psychiatric symptoms [50]. It also provided protection for individuals who had experienced trauma; reducing the risk of developing posttraumatic stress disorder and depressive symptoms [22, 48, 51–53]. Taken together, research suggests that CF serves as an important resilience factor, moderating the impact of various stressors on psychopathology symptoms.

Understanding the moderating role of CF in the relationship between SA and depressive symptoms may be illustrated using a real-life example: an individual with elevated SA symptoms may perceive themselves as inferior and boring [54, 55]; if they are low in CF, they will struggle to revise their beliefs about themselves and others, even when new information is available. This inflexibility might leave them with a negative perception and expectations of themselves and the world, thus fostering depressive symptoms [56, 57]. In contrast, a person high in CF may update their beliefs and interpretations smoothly [18, 19], acquiring more positive beliefs about the self and others, and in the long term, developing fewer depressive symptoms.

The current research findings may hold significant implications for the prevention of depressive symptoms among individuals with elevated levels of SA [58]. Approximately half of the individuals with SA will later develop depression [1]. However, a significant percentage of patients receiving empirically-validated treatments for SA continue to experience significant symptoms even after completing therapy [59]. As such, individuals who undergo treatment for SA may still be at risk of developing depressive symptoms. Thus, identifying patients less responsive to SA treatment and providing CF-focused interventions may help reduce this risk. Although caution is needed when interpreting these findings in relation to clinical populations, recent studies investigating therapeutic interventions support the rationale for targeting CF. Indeed, CF was found to be improved following various therapeutic interventions [60, 61], and this improvement was associated with a decrease in depressive symptoms [62]. Taken together, the research suggests that interventions that address CF as a key component may help prevent depressive symptoms for SA patients with low CF.

Our findings need to be interpreted in light of their limitations. First, although the current research used a longitudinal design, we did not examine the causal status of CF in the development of depressive symptoms. Empirically enhancing or decreasing CF levels among high SA individuals can deepen our understanding of this causality. Second, the current longitudinal study evaluated the measures at only two time points, 2 years apart, providing a limited perspective on the trajectory of depressive symptoms across an extended duration. Expanding the number of assessment points and examining a longer period in future research could provide a more thorough exploration of the dynamic nature of the relationship between SA and depressive symptoms over time. Third, the sample size of Study 2 was based on data collected in a previous project and did not rely on a priori power estimation. As such, the study may have been underpowered to detect smaller effects, and its findings should be interpreted with appropriate caution. Fourth, due to the relatively small sample sizes, the current research did not control for several potentially relevant variables (e.g., comorbid psychiatric disorders; current or past psychiatric or psychological treatment; medication use). In addition, the partial moderation effect found in Study 1 may suggest that other potential moderators, not assessed in the current research, may play a role in the relationship between SA and depressive symptoms. Indeed, other moderators—such as acceptance of socially anxious thoughts [12] or maternal major depressive disorder [63]—also contribute to shaping the relations between SA and depressive symptoms. Furthermore, due to the online nature of the study, we did not assess participants' diagnostic status. Future research would benefit from larger samples and in-person assessments to further elucidate the complex relationship between CF, SA, and depressive symptoms, controlling for other moderators and variables. Lastly, the current research used self-report measures, which align with other studies that measured CF (e.g., [64]). Future studies may aim to use a performance-based paradigm to support further the relationship between SA and the tendency to develop comorbid depressive symptoms [65]. Objective measures or a combination of self-report and behavioral assessments could strengthen the generalization of the findings.

### 4.1. Conclusions

Experiencing depressive symptoms usually coexists with other psychopathological symptoms, such as SA. This comorbidity creates a negative toxic cycle that impacts the lives of many individuals. The present research provided a preliminary insight into the role of CF as one possible mechanism that may contribute to this comorbidity and might foster the development of depressive symptoms. While more research is needed, such an understanding could eventually improve prevention efforts aimed at reducing the risk of depressive symptoms among individuals with elevated levels of SA.

## Figures and Tables

**Table 1:** Frequencies or means and standard deviations (in parentheses) of demographic characteristics and psychopathology severity by study.

Variable	Omega coefficient	Mean/frequencies	SD
Study 1:			
Demographic characteristics			
Gender (% females)	—	43.27	—
Age	—	38.26	11.02
Education	—	15.10	2.48
Psychopathology severity			
LSAS	0.97	45.96	29.75
CFS	0.90	53.89	9.16
BDI-II	0.97	9.82	11.83
% Individuals with SA symptoms above clinical cutoff (LSAS >50)	45.9	—	—
% Individuals with depressive symptoms above clinical cutoff (BDI-II >13)	29.8	—	—
Study 2:			
Demographic characteristics			
Gender (% females)	—	52.3	—
Age	—	39.41	11.17
Education	—	15.6	2.36
Psychopathology severity			
LSAS (T1)	0.98	46.43	29.77
CFS (T1)	0.92	56.44	8.49
BDI-II (T1)	0.97	8.63	10.32
% Individuals with SA symptoms above clinical cutoff (LSAS >50) (T1)	44.3	—	—
% Individuals with depressive symptoms above clinical cutoff (BDI-II >13) (T1)	33.8	—	—
LSAS (T2)	0.98	49.03	32.2
CFS (T2)	0.89	56.33	7.77
BDI-II (T2)	0.97	9.43	10.78
% Individuals with SA symptoms above clinical cutoff (LSAS >50) (T2)	19.8	—	—
% Individuals with depressive symptoms above clinical cutoff (BDI-II >13) (T2)	13.5	—	—

*Note:* LSAS = social anxiety symptoms; CFS = cognitive flexibility; BDI-II = depressive symptoms.

Abbreviations: T1, time 1; T2, time 2.

**Table 2:** Pearson correlations of age, education, social anxiety, cognitive flexibility, and depressive symptoms.

Variable	1	2	3	4	5	6	7	8
Study 1								
1. Age	—	—	—	—	—	—	—	—
2. Education	0.04	—	—	—	—	—	—	—
3. LSAS	−0.06	−0.10	—	—	—	—	—	—
4. CFS	19*⁣*^*∗∗∗*^	0.14*⁣*^*∗∗*^	−0.52*⁣*^*∗∗∗*^	—	—	—	—	—
5. BDI-II	−0.17*⁣*^*∗∗∗*^	−0.07	0.47*⁣*^*∗∗∗*^	−0.48*⁣*^*∗∗∗*^	—	—	—	—

**Variable**	**1**	**2**	**3**	**4**	**5**	**6**	**7**	**8**

At time 1 and time 2, Study 2								
1. Age	—	—	—	—	—	—	—	—
2. Education	0.06	—	—	—	—	—	—	—
3. LSAS (T1)	−0.09	−0.26*⁣*^*∗∗*^	—	—	—	—	—	—
4. CFS (T1)	0.02	0.14	−0.49*⁣*^*∗∗∗*^	—	—	—	—	—
5. BDI-II (T1)	−0.02	−0.12	0.49*⁣*^*∗∗∗*^	−0.63*⁣*^*∗∗∗*^	—	—	—	—
6. LSAS (T2)	−0.06	−0.18	0.81*⁣*^*∗∗∗*^	−0.43*⁣*^*∗∗∗*^	0.58*⁣*^*∗∗∗*^	—	—	—
7. CFS (T2)	0.03	0.09	−0.54*⁣*^*∗∗∗*^	0.75*⁣*^*∗∗∗*^	−0.57*⁣*^*∗∗∗*^	−0.52*⁣*^*∗∗∗*^	—	—
8. BDI-II (T2)	−0.07	−0.08	0.49*⁣*^*∗∗∗*^	−0.49*⁣*^*∗∗∗*^	0.75*⁣*^*∗∗∗*^	0.62*⁣*^*∗∗∗*^	−0.59*⁣*^*∗∗∗*^	—

*Note:* LSAS = social anxiety symptoms; CFS = cognitive flexibility; BDI-II = depressive symptoms.

*⁣* ^*∗∗*^ *p* < 0.01.

*⁣* ^*∗∗∗*^ *p* < 0.001.

**Table 3:** Hierarchical multiple regression analysis predicting depressive symptoms.

Variables	*B*	SE *B*	*T*	*β*	*R* ^2^	*ΔR* ^2^
Study 1						

Step 1	—	—	—	—	0.298	0.298*⁣*^*∗∗∗*^
Constant	9.82*⁣*^*∗∗∗*^	0.51	19.24	—	—	—
LSAS	0.12*⁣*^*∗∗∗*^	0.02	5.84	0.30*⁣*^*∗∗∗*^	—	—
CFS	−0.42*⁣*^*∗∗∗*^	0.07	−6.49	−0.33*⁣*^*∗∗∗*^	—	—
Step 2	—	—	—	—	0.306	0.008*⁣*^*∗*^
Constant	9.30*⁣*^*∗∗*^	0.57	16.32	—	—	—
LSAS	0.11*⁣*^*∗∗∗*^	0.02	5.5	0.28*⁣*^*∗∗∗*^	—	—
CFS	−0.44*⁣*^*∗∗∗*^	0.07	−6.71	−0.34*⁣*^*∗∗∗*^	—	—
LSAS*⁣*^*∗*^CFS	−0.004*⁣*^*∗*^	0.001	−2.02	−0.09*⁣*^*∗*^	—	—

At time 2, Study 2						

Step 1	—	—	—	—	0.326	0.326*⁣*^*∗∗∗*^
Constant	9.42*⁣*^*∗∗∗*^	0.86	10.96	—	—	—
LSAS (T1)	0.12*⁣*^*∗∗∗*^	0.03	3.57	0.33*⁣*^*∗∗∗*^	—	—
CFS (T1)	−0.42*⁣*^*∗∗∗*^	0.12	−3.62	−0.33*⁣*^*∗∗∗*^	—	—
Step 2	—	—	—	—	0.394	0.07*⁣*^*∗∗∗*^
Constant	8.13*⁣*^*∗∗∗*^	0.90	9.02	—	—	—
LSAS (T1)	0.11*⁣*^*∗∗*^	0.03	3.38	0.30*⁣*^*∗∗*^	—	—
CFS (T1)	−0.42*⁣*^*∗∗∗*^	0.11	−3.75	−0.32*⁣*^*∗∗∗*^	—	—
LSAS*⁣*^*∗*^CFS (T1)	−0.01*⁣*^*∗∗∗*^	0.003	−3.43	−0.26*⁣*^*∗∗∗*^	—	—

*Note:* LSAS = social anxiety symptoms; CFS = cognitive flexibility.

*⁣* ^*∗*^ *p*  < 0.05.

*⁣* ^*∗∗*^ *p*  < 0.01.

*⁣* ^*∗∗∗*^ *p*  < 0.001.

## Data Availability

The data and full code are publicly available at: https://osf.io/f93xz/?view_only=ef9d73087a744c189019cda0fe2dbec6.

## Supporting Information

Supporting Information Table S1 presents a hierarchical multiple linear regression of SA, CF, and their interaction on depressive symptoms, controlling for age. Table S2 presents demographic characteristics. Table S3 presents hierarchical multiple linear regression of depressive symptoms, SA, and CF at baseline, as well as SA × CF interaction in predicting depressive symptoms at follow-up, controlling for baseline depressive symptoms. Table S4 presents a cross-lag panel model predicting depressive symptoms at follow-up from depressive symptoms, SA, CF, and the interaction between SA and CF at baseline.
