# Supplementary material for: Temporal Dynamics of Social Anxiety and Depressive Symptoms: The Moderating Role of Cognitive Flexibility
Source: Depress Anxiety. 2025 Sep 15;2025:3055803. doi: 10.1155/da/3055803 (PMC12453920; doi:10.1155/da/3055803)
Supplement: Supporting Information — Table S1 presents a hierarchical multiple linear regression of SA, CF, and their interaction on depressive symptoms, controlling for age. Table S2 presents demographic characteristics. Table S3 presents hierarchical multiple linear regression of depressive symptoms, SA, and CF at baseline, as well as SA × CF interaction in predicting depressive symptoms at follow-up, controlling for baseline depressive symptoms. Table S4 presents a cross-lag panel model predicting depressive symptoms at follow-up from depressive symptoms, SA, CF, and the interaction between SA and CF at baseline. [file 3055803.f1.docx]

**Supplementary materials**

Study 1:

To examine the research hypothesis, we conducted a moderation analysis (model 1) using the PROCESS macro for R. Based on statistical guidelines (Henseler & Chin, 2010; Memon et al., 2019), independent measures were centered prior to the analysis. Table S1 presents hierarchical multiple linear regression of SA, CF, and their interaction on depressive symptoms severity, controlling for age. Results revealed that CF was marginally significant in moderating the relationship between SA and depressive symptoms $(F (1, 374)=3.44, p =.065)$.

Study 2:

Demographic characteristics are presented in Table S2.

To examine the research hypothesis, we conducted a hierarchical multiple linear regression of depressive symptoms, SA and CF at baseline, and well as SA*CF interaction in predicting depressive symptoms severity at follow-up, controlling for baseline depressive symptoms. (see Table S3). We conducted a moderation analysis (model 1) using the PROCESS macro for r. Based on Henseler & Chin, 2010 and Memon et al., 2019, independent measures were centered previous to the analysis. Results revealed that CF was marginally significant in moderating the relationship between SA and depressive symptoms $(F (1, 103)=3.07, p=.08)$. Subsequent simple slopes analyses indicated that for individuals reporting low levels of CF, higher levels of social anxiety were moderately associated with higher levels of depressive symptoms $(\beta=0.28, t=2.84, p=.005)$. In contrast, for individuals reporting high levels of CF, no association between SA and depressive symptoms was found $(\beta=0.04, t=0.4, p=.68)$.

We also performed a cross-lag panel model analysis in order to account for natural fluctuations across all variables. We first examined whether SA and CF predicted depressive symptoms at time 2 while controlling for baseline depressive symptoms (see Table S4) We then examined whether CF moderated the relationship between baseline SA and depressive symptoms at time 2. We conducted these analyses using the LAVAAN package in r. Overall, results revealed that CF was marginally significant in moderating the relationship between SA and depressive symptoms $(\beta= -.12, p=.057)$ suggesting a potential moderating effect of cognitive flexibility on the relationship between social anxiety and later depressive symptoms.

**Table S1**

*Hierarchical Multiple Regression Analysis Predicting Depressive Symptoms in* *Study 1*

| Variables | *B* | *SE B* | T | β | R^2^ | ΔR^2^ |
| --- | --- | --- | --- | --- | --- | --- |
| Step 1 |  |  |  |  | .306 | .306^***^ |
| Constant | 9.82*** | 0.51 | 19.33 |  |  |  |
| Age | -0.10* | 0.05 | -2.12 | -.09* |  |  |
| LSAS | 0.12*** | 0.02 | 5.94 | .30*** |  |  |
| CFS | -0.40 | 0.07 | -6.03 | -.31*** |  |  |
| Step 2 |  |  |  |  | .312 | 0.006^ |
| Constant | 9.34^***^ | 0.57 | 16.44 |  |  |  |
| Age | -0.09^ | 0.05 | -1.96 | -0.09^ |  |  |
| LSAS | 0.11^***^ | 0.02 | 5.6 | 0.29^***^ |  |  |
| CFS | -0.42^***^ | 0.07 | -6.24 | -0.32^***^ |  |  |
| LSAS*CFS | -0.003^*^ | 0.002 | -1.85 | -0.08^ |  |  |

*Note*. LSAS = social anxiety, CFS = cognitive flexibility

^^^p < .07. ^*^p < .05. ^**^ p < .01. ^***^ p < .001.

**Table S2**

*Frequencies or Means and Standard Deviations (in parentheses) of Demographic Characteristics and Psychopathology Severity of Participants who Took Part Only at Baseline, Study 2.*

| Variable | Mean / Frequencies | SD |
| --- | --- | --- |
| Demographic Characteristics |  |  |
| Gender (% females) | 52.3 |  |
| Age | 36.94 | 10.25 |
| Education | 15.28 | 2.08 |
| Psychopathology severity |  |  |
| LSAS | 49.22 | 28.9 |
| CFS | 53.81^*^ | 9.21 |
| BDI-II | 12.81^**^ | 12.15 |

*Note*. LSAS = social anxiety symptoms; CFS = cognitive flexibility; BDI-II = depressive symptoms.

^*^*p* < .05, ^**^*p* < .01.

*Independent t-test results revealed that participants who took part only at baseline reported higher levels of depressive symptoms and lower levels of CF as compared to those who took part at both the baseline and follow-up.

**Table S3**

*Hierarchical Multiple Regression Analysis Predicting Depressive symptoms at Time 2, from Depressive symptoms, Social Anxiety, Cognitive Flexibility, and the interaction between them at Time 1, Study 2.*

| Variables | *B* | *SE B* | T | β | R^2^ | ΔR^2^ |  |
| --- | --- | --- | --- | --- | --- | --- | --- |
| Step 1 |  |  |  |  | .588 | .588^***^ |  |
| Constant | 9.42^***^ | 0.68 | 13.95 |  |  |  |  |
| BDI-II (TI) | 0.72^***^ | 0.09 | 8.14 | 0.69^***^ |  |  |  |
| LSAS (T1) | 0.06^*^ | 0.03 | 2.21 | 0.17^*^ |  |  |  |
| CFS (T1) | 0.03 | 0.11 | 0.31 | 0.03 |  |  |  |
| Step 2 |  |  |  |  | .600 | 0.012 |  |
| Constant | 8.86^***^ | 0.74 | 11.92 |  |  |  |  |
| BDI-II (TI) | | 0.67^***^ | 0.09 | 7.27 | 0.64^***^ |  |  |
| LSAS (T1) | 0.16^*^ | 0.07 | 2.21 | 0.16^*^ |  |  |  |
| CFS (T1) | 0.004 | 0.11 | 0.04 | 0.01 |  |  |  |
| LSAS*CFS (T1) | -0.004^ | 0.002 | -1.75 | -0.12^^^ |  |  |  |

*Note*. LSAS = social anxiety symptoms, CFS = cognitive flexibility

^^^p < .09. ^*^p < .05. ^**^ p < .01. ^***^ p < .001.

**Table S4**

*Cross-lag panel model predicting Depressive symptoms at Time 2 from depressive symptoms, SA, CF, and the interaction between SA and CF at Time 1*

| Variables | *B* | *SE B* | z | β |
| --- | --- | --- | --- | --- |
| Step 1 |  |  |  |  |
| BDI | 0.72^***^ | 0.09 | 8.29 | 0.69^***^ |
| LSAS | 0.06^*^ | 0.03 | 2.26 | 0.17^*^ |
| CFS | 0.03 | 0.11 | 0.32 | 0.03 |
| Step 2 |  |  |  |  |
| BDI | 0.67^***^ | 0.09 | 7.83 | 0.66^***^ |
| LSAS | 0.06^*^ | 0.03 | 2.26 | 0.17^*^ |
| CFS | 0.004 | 0.10 | 0.04 | 0.003 |
| LSAS*CFS | -0.005^^^ | 0.002 | -1.90 | -0.12^^^ |

*Note*. LSAS = social anxiety symptoms, CFS = cognitive flexibility

^^^p < .07. ^*^p < .05. ^**^ p < .01. ^***^ p < .001.
